# Protective Effect of Antenatal Antioxidant on Nicotine-Induced Heart Ischemia-Sensitive Phenotype in Rat Offspring

**DOI:** 10.1371/journal.pone.0150557

**Published:** 2016-02-26

**Authors:** DaLiao Xiao, Lei Wang, Xiaohui Huang, Yong Li, Chiranjib Dasgupta, Lubo Zhang

**Affiliations:** 1 Center for Perinatal Biology, Department of Basic Sciences, Loma Linda University School of Medicine, Loma Linda, California, United States of America; 2 Department of Traditional Chinese Medicine, Shanghai Putuo District People’s Hospital, Shanghai, PR China; Mount Sinai School of Medicine, UNITED STATES

## Abstract

Fetal nicotine exposure increased risk of developing cardiovascular disease later in life. The present study tested the hypothesis that perinatal nicotine-induced programming of heart ischemia-sensitive phenotype is mediated by enhanced reactive oxygen species (ROS) in offspring. Nicotine was administered to pregnant rats *via* subcutaneous osmotic minipumps from day 4 of gestation to day 10 after birth, in the absence or presence of a ROS inhibitor, N-acetyl-cysteine (NAC) in drinking water. Experiments were conducted in 8 month old age male offspring. Isolated hearts were perfused in a Langendorff preparation. Perinatal nicotine treatment significantly increased ischemia and reperfusion-induced left ventricular injury, and decreased post-ischemic recovery of left ventricular function and coronary flow rate. In addition, nicotine enhanced cardiac ROS production and significantly attenuated protein kinase C_ε_ (PKC_ε_) protein abundance in the heart. Although nicotine had no effect on total cardiac glycogen synthase kinase-3β (GSK3β) protein expression, it significantly increased the phosphorylation of GSK3β at serine 9 residue in the heart. NAC inhibited nicotine-mediated increase in ROS production, recovered PKC_ε_ gene expression and abrogated increased phosphorylation of GSK3β. Of importance, NAC blocked perinatal nicotine-induced increase in ischemia and reperfusion injury in the heart. These findings provide novel evidence that increased oxidative stress plays a causal role in perinatal nicotine-induced developmental programming of ischemic sensitive phenotype in the heart, and suggest potential therapeutic targets of anti-oxidative stress in the treatment of ischemic heart disease.

## Introduction

Tobacco smoking is one of the leading causes of preventable morbidity and mortality worldwide, accounting for nearly $200 billion in health care costs each year within the United States alone [1]. Recently, epidemiological studies have clearly shown an increased risk of cardiovascular disease in children born to women who smoked during pregnancy [2–5]. As one of the major active components in cigarettes, nicotine may contribute to maternal smoking-induced developmental programming of cardiovascular dysfunction in offspring. Indeed, fetal nicotine exposure during pregnancy caused cardiovascular disorders later in life in several different animal models [6–8]. Furthermore, recent studies have shown that exposure to electronic cigarette (e-cigarette), a nicotine delivery system, causes a cardiac development defect in zebrafish and human embryonic stem cells *in vivo* and *in vitro* [9]. Previous studies in our laboratory demonstrated that perinatal nicotine exposure reprogrammed cardiovascular function and caused a development of heart ischemia-sensitive phenotype in adult offspring [10–15]. However, the underlying molecular mechanisms are largely unknown.

In present study we sought to explore the mechanistic link between perinatal nicotine exposure and the aberrant development of heart ischemia-sensitive phenotype. One of the vital mechanisms that contributes to myocardial ischemia/reperfusion injury and heart dysfunction is oxidative stress. Reactive oxygen species (ROS) are well-recognized as key signaling molecules that mediate diverse biological responses. In the cardiovascular system ROS have a significant pathophysiological role in cardiovascular remodeling and dysfunction [16–18]. There is increasing evidence that altered ROS production may be critically important in the fetal programming of cardiovascular disorders in adulthood [19–20]. Studies in different animal models have demonstrated that enhanced ROS production plays a role in altering the cardiac function of developmentally programmed animals [21–22]. In addition, the treatment with antioxidants significantly improved recovery following ischemia and reperfusion in the programmed hearts [23]. Maternal smoking is associated with increased levels of ROS in offspring [24]. Furthermore, exposure to nicotine during gestation results in increased ROS in fetal, neonatal and adult tissues [25, 26]. Recently, our studies have shown that perinatal nicotine exposure induces a fetal programming of adult hypertensive phenotype associated with a heightened ROS production in vasculatures [12–13, 27]. These studies suggest that nicotine-mediated changes of ROS production in cardiac tissues may explain the increased susceptibility of the heart to ischemia/reperfusion (I/R) injury.

We have previously shown that adult rat hearts born after a perinatal nicotine exposure have an increased susceptibility to ischemia/reperfusion (I/R) [14, 15]. Here we take a position to test a novel hypothesis that increased oxidative stress plays a causal role in perinatal nicotine-mediated programming of heart ischemia-sensitive phenotype in adult offspring. To test the hypothesis, first we examined whether perinatal nicotine exposure would increase cardiac ROS production and whether antenatal antioxidant would block nicotine-mediated heightened ROS production in adult hearts. Then we evaluated ex vivo whole organ function in adult hearts between the saline control and perinatal nicotine-exposed rat offspring to see whether antenatal antioxidant would inhibit nicotine-mediated heart I/R injury and heart dysfunction in adult offspring. In addition, we examined the effect of antenatal antioxidant treatment on cardiac signaling protein and ischemia protective protein expression in both the saline control and nicotine-treated offspring.

## Materials and Methods

### Ethics Statement

The Institutional Animal Care and Use Committee of Loma Linda University had approved this study (IACUC# 8140052). Rats used in this study were euthanized by inhalation of 5% isoflurane, followed by removal of their hearts. All procedures and protocols followed the guidelines in the National Institutes of Health Guide for the Care and Use of Laboratory Animals.

### Experimental animals

Time-dated (day 2 of gestation) pregnant Sprague-Dawley rats were purchased from Charles River Laboratories (Portage, MI) and housed individually in Plexiglas acrylic plastic cages in air-conditioned rooms (room temperature 22°C, relative humidity 60%; lights on from 8:00 a.m. to 8:00 p.m.). Pellet food and tap water were available *ad libitum*. On day 4 of gestation, the rats were randomly divided into four groups: 1) saline control (n = 5), 2) nicotine (n = 5), 3) saline plus the ROS inhibitor, N-acetyl-cysteine (NAC, 500 mg/kg/day) (n = 5), and 4) nicotine plus NAC (n = 6). Nicotine was administered to pregnant rats through an osmotic minipump at 4 μg/kg/min from day 4 of pregnancy to 10 days after birth, as described in detail previously [10–15, 27]. The dose of nicotine resulted in blood levels closely resembling those occurring in moderate human smokers [2]. Control rats received saline from the osmotic minpump as the vehicle control. For the antioxidant treatment, NAC was placed in the drinking water which was concurrently started at the time as nicotine infusion at day 4 of pregnancy and ended at the same time as nicotine treatment, as described in detail previously [27]. The concentration of NAC placed in the drinking water has shown to be effective at reducing oxidative stress [28]. Our previous studies [10, 11, 14] and current study did not show any significant difference in litter size following the nicotine exposure. Therefore, the litter size was intact as nature in each dam and all of the newborn pups were kept with their mothers until weaning. At weaning (3-weeks old), the male and female offspring were separated. Our previous studies have shown a gender-dependent increase in vascular reactivity in response to prenatal nicotine exposure in male offspring, therefore, the male offspring were kept and used for present studies at the age of 8 months.

### Measurement of cardiac function and ischemia and reperfusion injury

The hearts were isolated from the adult offspring and retrogradely perfused *via* the aorta in a modified Langendorff apparatus under constant pressure (70 mmHg) with gassed (95% O_2_, 5% CO_2_) Krebs-Henseleit buffer at 37°C, as described in detail previously [29]. A pressure transducer connected to a saline-filled balloon inserted into the left ventricle (LV) was used to assess ventricular function by measuring the ventricular pressure (mmHg) and its first derivative (dP/dt). LV end diastolic pressure (LVEDP) was set at approximately 5 mmHg. After baseline recording for 60 minutes, hearts were subjected to 25 minutes of global ischemia followed by 60 minutes of reperfusion. Left ventricular developed pressure (LVDP), heart rate (HR), dp/dt_max_, dp/dt_min_, and LV end-diastolic pressure (LVEDP) were continuously recorded. Myocardial infarct size was measured as described in detail previously [29]. In brief, at the end of reperfusion, left ventricles were collected, cut into four slices, incubated with 1% triphenyltetrazolium chloride solution for 15 minutes at 37°C, and immersed in formalin for 30 minutes. Each slice was then photographed separately, and the areas of myocardial infarction in each slice were analyzed by computerized planimetry, corrected for the tissue weight, summed for each heart, and expressed as a percentage of the total left ventricle weight. Lactate dehydrogenase (LDH) activity was measured in coronary effluent collected at the end of ischemia/reperfusion (I/R), using a TOX 7 assay kit from Sigma following the manufacturer’s instructions.

### Western blot analysis

Protein abundance of protein kinase C epsilon (PKCε), glycogen synthase kinase-3β (GSK3β), and phospho-GSK3β in the left ventricles of the heart was measured with western blot analysis as described previously [15, 28]. In brief, tissues were homogenized in a lysis buffer containing 150 mM NaCl, 50 mM Tris HCl, 10 mM EDTA, 0.1% Tween 20, 0.1% β-mercapto-ethanol, 0.1 mM phenylmethylsulfonyl fluoride, 5 μg/ml leupeptin, and 5 μg/ml aprotinin, pH 7.4. Homogenates were then centrifuged at 4°C for 10 min at 10,000g, and supernatants were collected. Samples with equal proteins were loaded on to the 7.5% polyacrylamide gel with 0.1% SDS and were separated by electrophoresis at 100 V for 90 minutes. Proteins were then transferred to the nitrocellulose membrane and incubated with primary antibodies for PKCε (Santa Cruz Biotechnology, Inc., Santa Cruz, CA), phospho-GSK3β (Ser 9), and total GSK3β (Cell Signaling, Danvers, MA), respectively. After washing, membranes were incubated with horseradish peroxidase-conjugated secondary antibodies (GE Healthcare, Chalfont St. Giles, Buckingghamshire, UK). Proteins were visualized with enhanced chemiluminescence reagents, and blots were exposed to Hyperfilm (GE Healthcare). Results were quantified with electrophoresis documentation and analysis. Band intensities were normalized to actin.

### Measurement of vascular ROS

Total ROS in the left ventricle isolated from the adult offspring were measured with the Oxiselect In Vitro ROS/RNS Assay Kit (Cell Biolabs, San Diego, CA), following the manufacturer’s instructions and described previously [29]. Briefly, tissues were homogenized in cold PBS solution and centrifuged at 10,000 g for 5 minutes. The supernatant was collected for the assay. 50 μL of unknown samples or standard were added to a 96-well plate and mixed with 50 μL of catalyst and 100 μL of 2’, 7’-dichlorodihydrofluorescein diacetate (DCF). After incubation at room temperature for 30 minutes, the fluorescence (Ex480nm/Em530nm) was measured using a Synergy HT Multi-Mode Microplate Reader (Bio-Tek Instruments, Inc., Winooski, VT, USA).

### Statistical analysis

Data were expressed as means ± SEM obtained from the number (n) of experimental animals given. Statistical significance (P < 0.05) was determined by analysis of variance (ANOVA) or Student’s *t*-test, where appropriate.

## Results

### Effect of antioxidant on nicotine-mediated increase in heart susceptibility to ischemic injury

**Fig 1:**
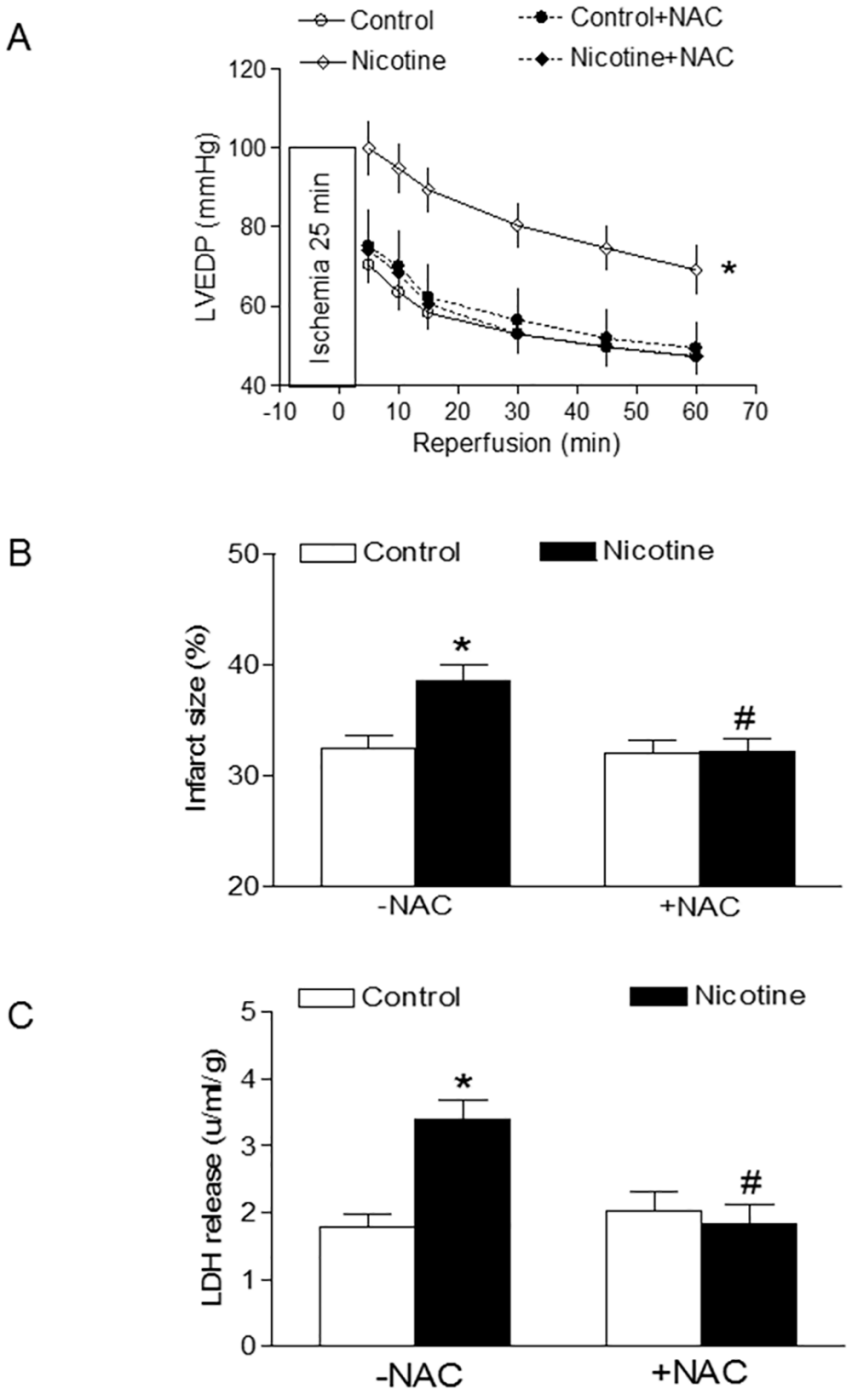
Effects of antenatal antioxidant on nicotine-induced increase in ischemia-reperfusion (I/R) injury in adult offspring. Hearts were isolated from the four groups of rats that were prenatally exposed to saline control, saline control plus NAC, nicotine, or nicotine plus NAC. Then the hearts were subjected to 25 min of ischemia and 60 min of reperfusion in a Langendorff preparation. Post-ischemic recovery of the left ventricular end-diastolic pressures (LVEDP) was determined during the course of reperfusion (**A**). Left ventricles were collected at the end of reperfusion, and myocardial infarct size was determined with 1% triphenyltrazolium chloride staining and expressed as a percentage of the total ventricular weight (**B**). Lactate dehydrogenase (LDH) activity was measured in coronary effluent collected at end of I/R (**C**) Data are means ± SEM of animals (n = 4 to 5 litters) from each group. Data were analyzed by 2-way ANOVA. *P < 0.05 vs. control, ^#^P < 0.05 vs. +NAC.

**Fig 2:**
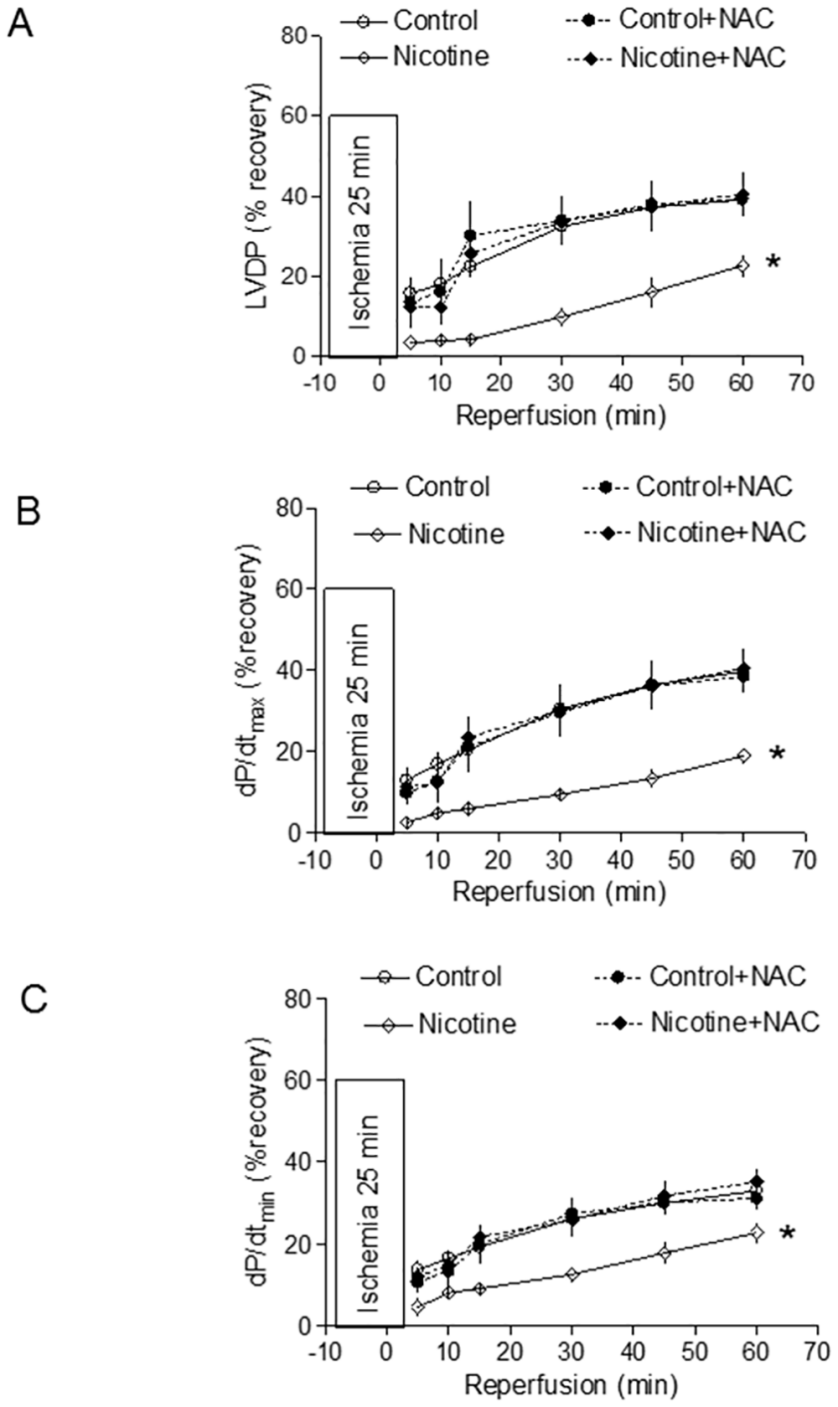
Effects of antenatal antioxidant on post-ischemic recovery of LV function in adult offspring. Hearts were isolated from the four groups of rats that were prenatally exposed to saline control, saline control plus NAC, nicotine, or nicotine plus NAC. Then the hearts were subjected to 25 min of ischemia and 60 min of reperfusion in a Langendorff preparation. Post-ischemic recoveries of the left ventricular diastolic pressures (LVDP) (**A**), dP/dt_max_ (**B**) and dP/dt_min_ (**C**) were determined during the course of reperfusion. Data are means ± SEM of animal numbers from each group. Data were analyzed by 2-way ANOVA. *P < 0.05 versus saline control for the entire curve.

Table 1 shows the pre-ischemic values of LV function and coronary flow rate in isolated hearts from the adult offspring in a Langendorff preparation. Perinatal nicotine exposure showed no significant effects on baseline LV function and coronary flow of adult rat hearts both in the absence and presence of NAC treatment (Table 1). As shown in Figs 1 and 2, global ischemia for 25 minutes caused a significant damage of LV function in the rat offspring. In the absence of NAC, perinatal nicotine exposure caused a significant increase in LVEDP, myocardial infarct size, and LDH release of the heart after 25 minutes of ischemia and 60 minutes of reperfusion (Fig 1). However, in the presence of NAC (Fig 1), there were no significant differences in LVEDP, myocardial infarct size, and LDH release of hearts between the saline control and nicotine-treated group. As shown in Fig 2, the increased I/R-induced LV heart injury was associated with a decrease in post-ischemic recovery of LV function. In the absence of NAC, perinatal nicotine exposure resulted in significant decreases in post-ischemic recovery of LVDP and dP/dt_max_, and dP/dt_min_ in the hearts compared with the control (Fig 2). However, in the presence of NAC, there were no significant differences in the post-ischemic recovery of LV function between the saline control and nicotine-treated animals (Fig 2).

**Table 1:** Preischemic Left Ventricle Functional Parameters of 8 Month-old Male Offspring.

Animal group	n	LVDP (mmHg)	dP/dt_max_ (mmHg/s)	dP/dt_min_ (mmHg/s)	Coronary flow (ml/min/g)
**Saline**	7	101.6±3.2	3083±114	1925±109	7.6±0.21
**Nicotine**	6	96.0±3.8	2964±161	1790±207	7.1±0.37
**Saline + NAC**	7	101.9±2.5	3336±118	2006±99	8.0±0.46
**Nicotine + NAC**	7	100.3±3.3	3164±132	1861±47	7.6±0.50

**Fig 3:**
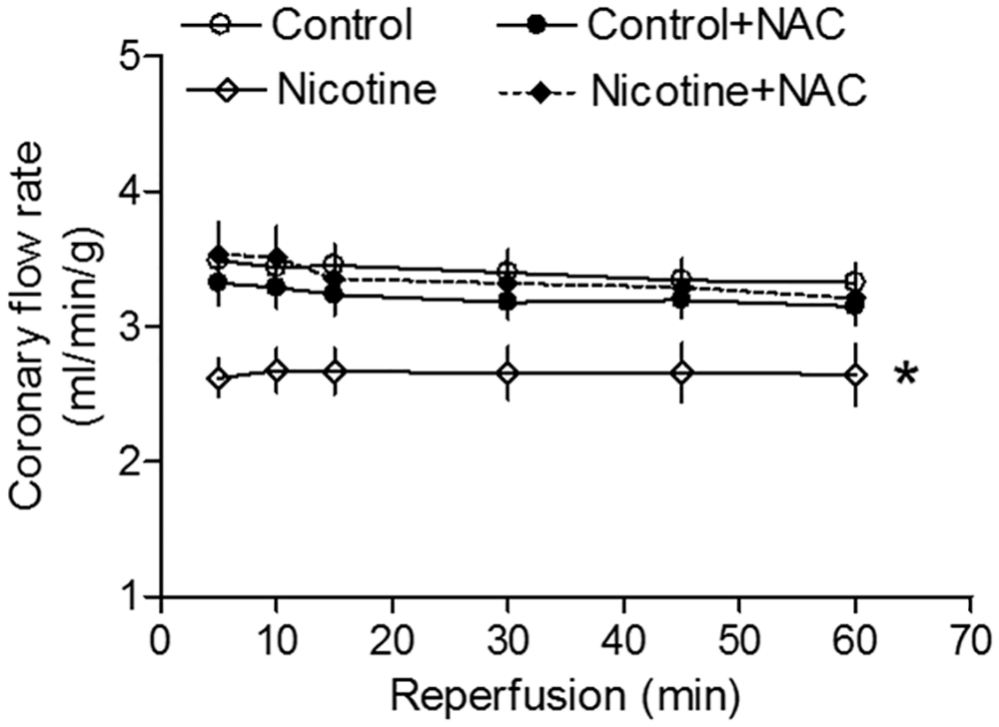
Effects of antenatal antioxidant on coronary flow rate in adult offspring. Hearts were isolated from the four groups of rats that were prenatally exposed to saline control, saline control plus NAC, nicotine, or nicotine plus NAC. Then the hearts were subjected to 25 min of ischemia and 60 min of reperfusion in a Langendorff preparation. Pulmonary artery effluent was collected as an index of coronary flow (milliliters per minute per gram of heart wet weight). Data are means ± SEM of animal numbers from each group. Data were analyzed by 2-way ANOVA. *P < 0.05 versus saline control for the entire curve.

Fig 3 shows the effect of NAC on perinatal nicotine-mediated coronary flow. In the absence of NAC, perinatal nicotine exposure significantly decreased coronary flow rate during post-ischemic recovery in the adult offspring. However in the presence of NAC, there was no significant difference in coronary flow rate between the saline control and nicotine-treated groups.

### Antioxidant blocked nicotine-mediated increase in ROS production

**Fig 4:**
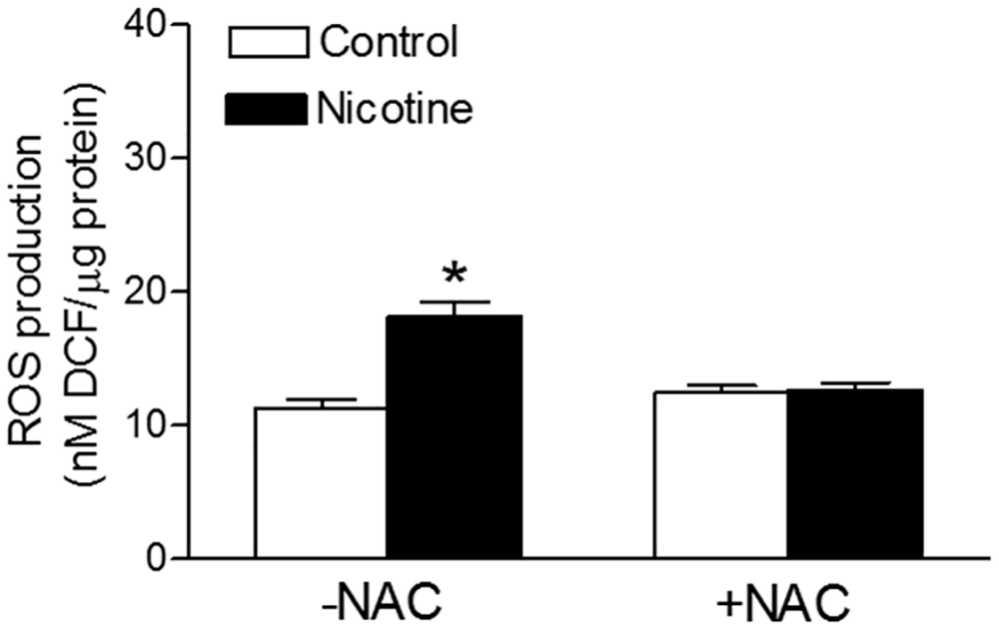
Effects of antenatal antioxidant on reactive oxygen species (ROS) production in hearts. Hearts were isolated from adult offspring that were prenatally exposed to saline control or nicotine along without or with NAC treatment. Total ROS production in the hearts was measured with 2’,7’-dichlorodihydrofluorescein (DCF)-based quantitative assay kits. All of the data expressed as means ± SEM of animal numbers per group. Data were analyzed by 2-way ANOVA, followed by Bonferroni post-tests. *P < 0.05 vs. control.

In the absence of NAC, the perinatal nicotine treatment resulted in a significant increase in ROS productions in the LV heart of adult offspring, as compared with the saline control animals (Fig 4). In the presence of NAC, there was no significant difference in ROS production between the two groups (Fig 4). In addition, NAC treatment did not significantly affect ROS production in control offspring.

### Antioxidant abrogated nicotine-mediated decrease in PKCε expression

**Fig 5:**
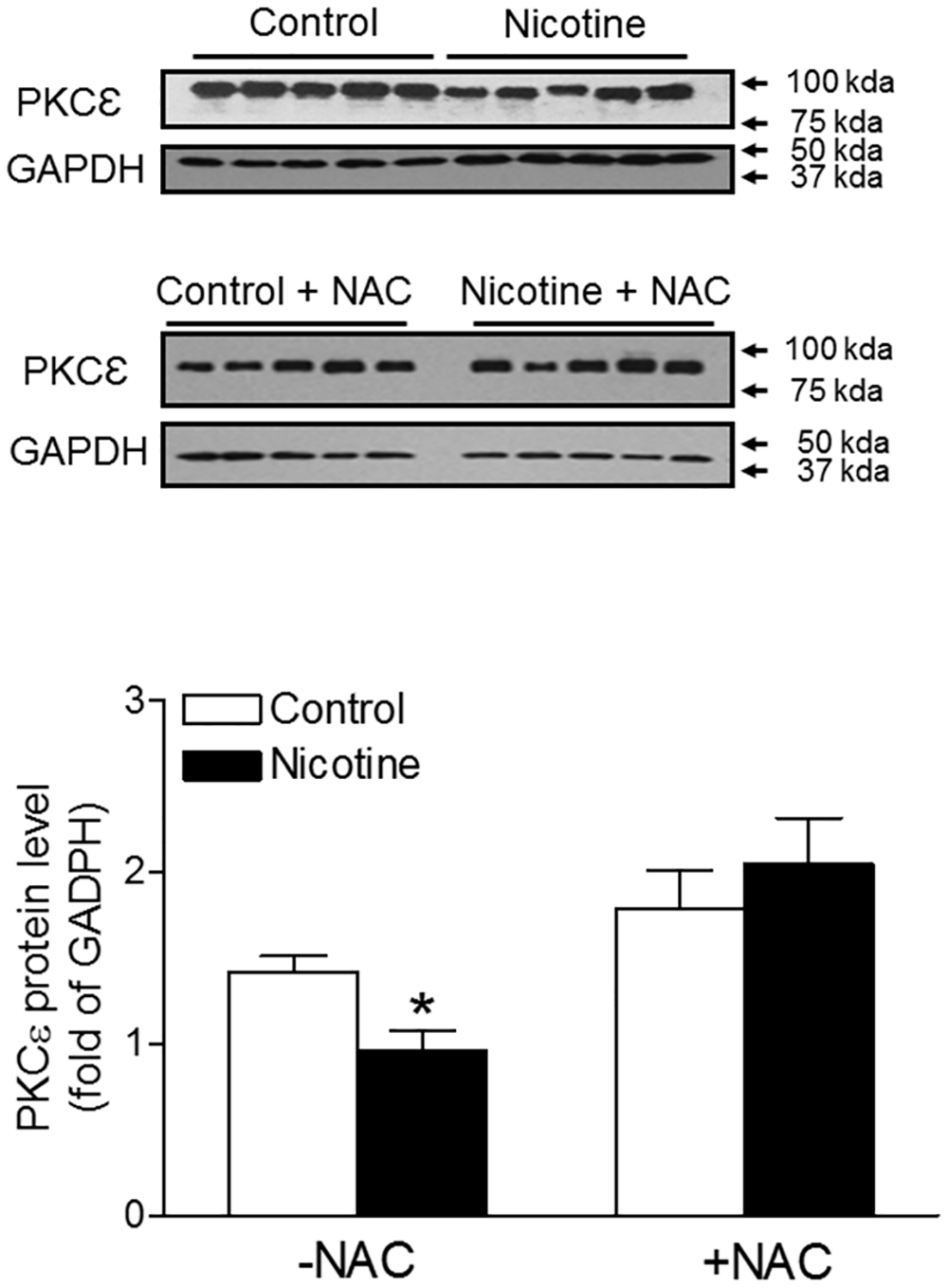
Effects of antenatal antioxidant on PKC_ε_ protein levels in the hearts of adult offspring. Hearts were isolated from adult offspring that were prenatally exposed to saline control or nicotine along without or with NAC treatment. PKC_ε_ protein levels in the hearts were determined by Western blots. The protein density was normalized to GAPDH. All of the data expressed as means ± SEM of animal numbers per group. Data were analyzed by Student’s *t*-test. *P < 0.05 vs. control.

PKCε gene expression pattern has a significant impact in the regulation of heart hypertrophy and PKCε activation plays a vital role in cardio-protection in the setting of heart ischemia and reperfusion injury. As shown in Fig 5, perinatal nicotine exposure caused a significant decrease in PKCε protein abundance in the hearts as compared with the saline control groups. However, the difference in PKCε protein abundance between the saline control and nicotine-treated groups was abolished by NAC treatment.

### Antioxidant abrogated nicotine-mediated increase in GSK3β activity

**Fig 6:**
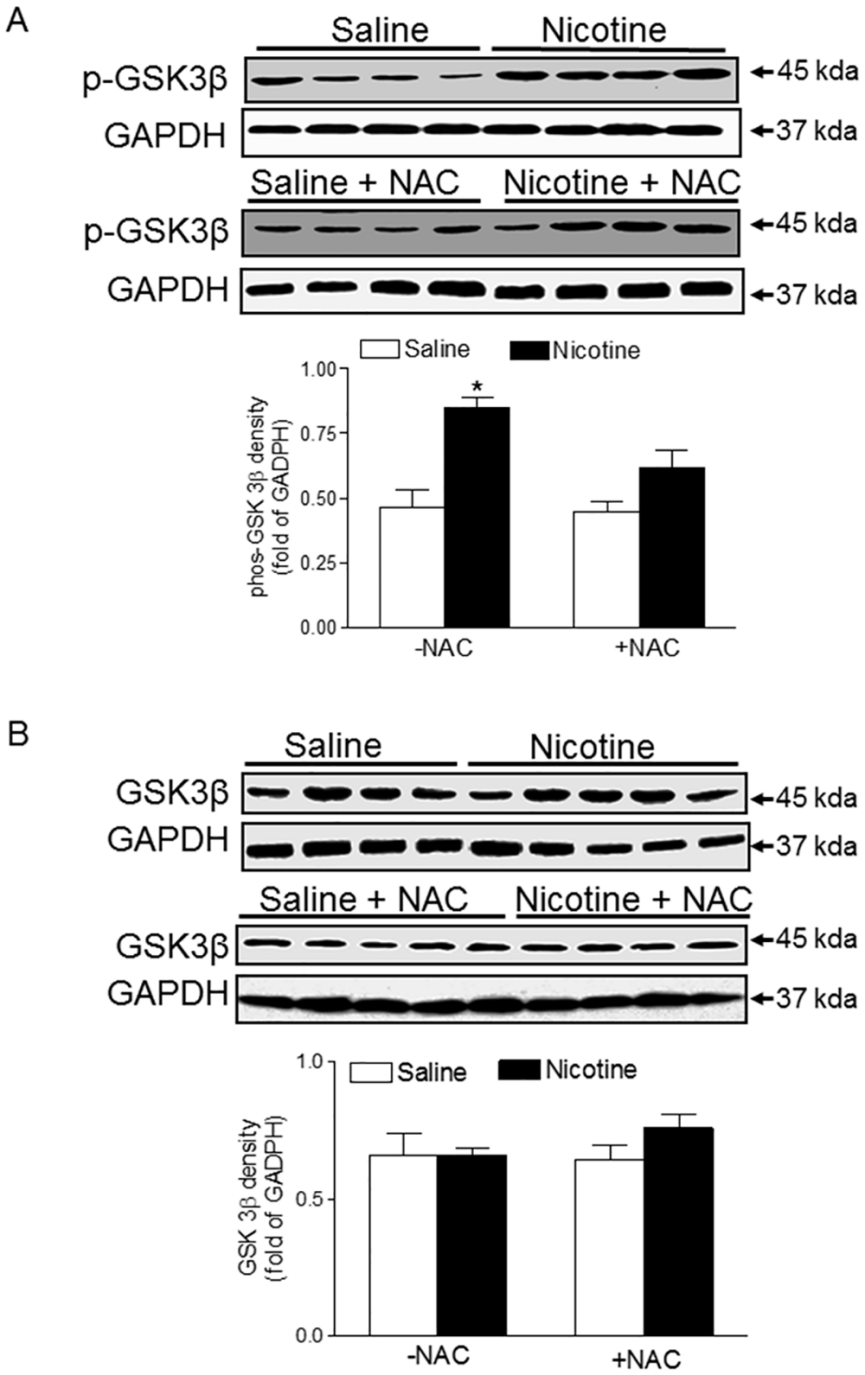
Effects of antenatal antioxidant on GSK3β gene expression and its activity in the hearts of adult offspring. Hearts were isolated from adult offspring that were prenatally exposed to saline control or nicotine along without or with NAC treatment. Phospho-GSK3β (**A**) and total GSK3β protein (**B**) levels in the hearts were determined by Western blots. The protein density was normalized to GAPDH. All of the data expressed as means ± SEM of animal numbers per group. Data were analyzed by Student’s *t*-test. *P < 0.05 vs. saline control.

Glycogen synthase kinase-3β (GSK3β) is a master regulator of cardiac cell growth and death and thus is involved in the setting of heart ischemic injury. To investigate the role of GSK3β signaling in mediating cardiac dysfunction in response to perinatal nicotine exposure, the phosphorylation levels of GSK3β at serine 9 and GSK3β protein expression were determined by Western blot analysis. As shown in Fig 6, perinatal nicotine exposure had no significant effect on GSK3β protein expression, but enhanced serine 9 phosphorylation of GSK3β in the hearts as compared with the saline control. However, NAC treatment significantly halted nicotine-induced alteration of serine 9 phosphoryltion level of GSK3β and eliminated the difference between the saline control and nicotine-treated animals.

## Discussion

The present study provides direct evidence that nicotine-mediated increased oxidative stress plays a causal role in the fetal programming of ischemic sensitive phenotype of heart later in life. The major findings in the present study are: (1) perinatal nicotine exposure significantly increased LV myocardial infarct size and decreased post-ischemic recovery of LV function after global ischemia in adult offspring, which was associated with an increased ROS production in the hearts; (2) antenatal antioxidant treatment prevented nicotine-mediated increase in ischemia/reperfusion-induced heart injury and improved post-ischemic recovery of LV function in the offspring; (3) perinatal nicotine exposure significantly decreased coronary flow rate as compared with the saline control group during post-ischemic recovery in the adult hearts, which was reversed by antenatal antioxidant treatment; (4) perinatal nicotine exposure suppressed cardiac PKC_ε_ expression as compared with the saline treated group. However, antenatal antioxidant treatment eliminated the difference in cardiac PKC_ε_ expression between the nicotine-treated and saline control groups; (5) perinatal exposure to nicotine had no effect on total cardiac GSK3β protein expression but significantly enhanced the phosphorylation of GSK3β as compared with the saline control, which was eliminated by antenatal antioxidant treatment.

Previous studies have shown that maternal smoking/nicotine use during pregnancy increases the biomarkers of oxidative stress in infants [24] and ROS production in vasculatures and pancreatic tissues of offspring [12, 25, 30]. Consistent with the previous studies, the present study showed an increase in cardiac ROS production in offspring that had been prenatally exposed to nicotine. Although the exact mechanisms underlying nicotine increasing oxidative stress are not completely clear, our previous studies have shown that perinatal nicotine-mediated increased ROS production is associated with an increase in NADPH oxidase 2 (NOX2) expression in vasculatures [12] and NOX1 expression in cardiomyocytes [31]. These findings suggest that NADPH oxidase-dependent oxidative stress may be one of the key molecular mechanisms in nicotine-mediated heightened ROS production. NAC is a potent free radical scavenger with antioxidant properties that has been used in the clinical setting and animal models [32–34]. In the present study our data showed that the nicotine-mediated increased ROS in adult hearts were eliminated by maternal NAC treatment. This suggests that NAC is capable of decreasing nicotine-induced cardiac oxidative stress. These findings are also consistent with previous reports that maternal NAC treatment inhibits fetal stress-induced ROS production in fetus and adult offspring [27, 35, 36], and show an *in utero* benefit of NAC treatment in pregnancies complicated by nicotine exposure. However, the effect of NAC in the treatment of functional disorders such as ischemia-reperfusion cardiac injury and liver hepatitis may be regulated through other mechanisms besides scavenging free radicals [37, 38]. It has been reported that NAC can improve the histopathological changes and suppress inflammatory cytokines in concanavalin A-induced hepatitis [38]. Therefore, in our future studies, we will need to employ a specific ROS scavenger or antioxidant to confirm whether the heightened ROS is the key mechanism underlying nicotine-induced heart ischemia-sensitive phenotype in offspring.

The present study showed that perinatal nicotine exposure had no effect on pre-ischemic baseline values of heart function but significantly increased the LV myocardiac infarct size and decreased post-ischemic recovery of LV function after 25 minutes of ischemia/reperfusion in 8 month-old adult offspring. This finding is in agreement with previous studies showing that perinatal nicotine exposure increased cardiac vulnerability to I/R injury in 3-month-old offspring [14]. The molecular mechanisms underlying perinatal nicotine-mediated increased heart ischemic injury and heart dysfunction in offspring are largely unclear. Evidence has shown that an increase in fetal oxidative stress results in cardiovascular dysfunction in response to intrauterine adverse environmental exposure in different animal models [19, 20, 39, 40]. In the present study, we found that maternal antioxidant treatment significantly improved I/R-induced injury and the post-ischemic recovery in cardiac function of perinatal nicotine-treated offspring. The finding provides direct evidence that nicotine-mediated heightened oxidative stress may be one of the vital molecular mechanisms underlying fetal programming of adult heart ischemia-sensitive phenotype. Similarly, previous studies have reported that antioxidant intervention to nicotine-exposed dams prevents the pancreas β-cell loss and apoptosis observed in nicotine-exposed male offspring [30]. In addition, we and others have demonstrated that antenatal antioxidant treatment prevents adult hypertension and vascular dysfunction associated with *in utero* exposure to nicotine [27] or to a low-protein diet [19]. Furthermore, a previous study has reported that treatment with antioxidant NAC significantly decreased the cardiac ischemia/reperfusion injury and improved the post-ischemic cardiac function in maternal low protein fed offspring [22]. Taken together, these findings suggest that perinatal oxidative stress may be one of the most common mechanisms underlying intrauterine adverse environmental stimuli-mediated increased risk of cardiovascular disease in adulthood.

Heart contractile function may also be confounded by the low coronary flow rate that occurs during ischemia and reperfusion. Our previous studies have demonstrated that nicotine-mediated attenuated post-ischemic recovery of LV function is associated with a gender-dependent decrease in coronary flow rates in female but not male offspring at the age of 3 months [14]. However, in the present study we found that post-ischemic coronary flow rates were significantly decreased in perinatal nicotine-exposed male offspring as compared with the saline control rats at the age of 8 months. These findings suggest that the effect of perinatal nicotine exposure on post-ischemic coronary flow rate in hearts is age and gender-dependent. This decrease in coronary perfusion, and subsequent decrease in the delivery of oxygen, may suppress cardiac contractile function and contribute to the nicotine-mediated post-ischemic recovery of heart function. A novel finding of the present study is that maternal NAC treatment significantly reversed the nicotine-induced decreased coronary flow rates in the adult hearts, which suggests that nicotine-mediated ROS may directly program coronary vascular dysfunction. This observation is consistent with previous reports that NAC improves coronary vascular function which improves flow into the coronary beds [41].

In addition to changes in coronary flow, intrinsic changes in cardiomyocytes play a major role in cardiac programming resulting from an adverse intrauterine environment [14, 15, 28, 42–44]. It has been clearly shown that PKC_ε_ gene, serving as an intrinsic cardio-protective protein, plays a pivotal role in cardio-protection during cardiac I/R injury [45–47]. Previous studies have demonstrated that antenatal insults such as hypoxia, cocaine and nicotine cause an epigenetic down-regulation of PKC_ε_ expression in the cardiomyocytes of offspring [14, 15, 44, 48]. The present findings that perinatal nicotine exposure significantly decreased cardiac PKC_ε_ protein expression in adult offspring, further suggest a common mechanism of PKC_ε_ in cardiac programming in response to intrauterine adverse stimuli. Although the epigenetic mechanisms underlying nicotine-mediated cardiac PKC_ε_ protein repression are not clear, previous studies suggest that increased methylation of CpG dinucleotide sequences in the promoter region of PKC_ε_ gene may be one of the key epigenetic mechanisms [15, 48, 49]. Of importance, increasing evidence suggests that increased ROS may lead to the alteration of DNA methylation status, resulting in epigenetic regulation of gene expression patterns [50–52]. A previous study has provided direct evidence that inhibition of ROS with NAC prevented the increase in global DNA methylation and concomitant increased DNA methyltransferases (Dnmt) expression in melanoma cell lines [53], which suggests that oxidative stress may directly regulate DNA methylation through Dnmt expression modification. Our recent studies have demonstrated that norepinephrine causes epigenetic repression of the PKC_ε_ gene in rodent hearts through ROS signaling pathway [31]. Consistent with the previous studies, the present study showed that antenatal antioxidant treatment reversed nicotine-mediated decreased cardiac PKC_ε_ gene expression. Taken together, these findings suggest that nicotine-mediated oxidative stress may increase DNA methylation at the PKC_ε_ promoter region, resulting in down-regulation of PKC_ε_ gene expression in the heart.

Similar to PKCε gene, GSK3β gene is another one of the central regulators of embryonic cardiomyocyte proliferation and differentiation. Increasing evidence shows that GSK3β serves as an essential negative regulator of cardiac hypertrophy and is involved in the setting of heart ischemic injury [54–56]. Therefore, inhibition of GSK3β is an important mechanism contributing to the development of cardiac dysfunction and heart failure. Unlike most protein kinases, GSK3β remains active in its dephosphorylated form and is inactivated upon phosphorylation of serine 9 residue by other factors [57, 58]. In the current study, our data indicated that perinatal nicotine increased phosphorylation of GSK3β at the serine 9 residue. It suggests that nicotine-mediated heightened serine 9 phosphorylation of GSK3β may inactivate cardiac GSK3β, leading to an increase in I/R-induced cardiac injury. Furthermore, our current finding that NAC treatment restored the serine 9 phosphorylation level of GSK3β and eliminated the difference between the saline control and nicotine-treated groups, suggests that the increased phosphorylation of GSK3β gene may be regulated through an oxidative stress-dependent mechanism. Similar findings have been reported in a diabetic animal model showing that antioxidant treatment causes an inactivation of GSK3β, leading to prevention of diabetic cardiomyopathy [59]. Although it remains unclear exactly how antioxidants inhibit GSK3β activation, one explanation is that ROS may directly regulate PKC_ε_ expression/activation, an upstream negative mediator of GSK3β [57]. Nonetheless, an investigation into the exact mechanism responsible is warranted for future study.

In conclusion, the present study provides novel evidence that perinatal nicotine-mediated increased oxidative stress is a pivotal epigenetic molecular regulator in fetal programming of ischemia-sensitive phenotype in adult hearts. Our data showed that perinatal nicotine exposure increased cardiac ROS production, which caused an epigenetic down-regulation of the cardioprotective protein, PKC_ε_ gene expression. In addition, the heightened ROS increased cardiac GSK3β phosphorylation, resulting in inactivation of the GSK3β gene. The nicotine-mediated decreased PKC_ε_ gene expression and the increased inactivated GSK3β form may lead to an increase in the heart I/R injury and dysfunction. Finally, the finding that antenatal antioxidant treatment reversed the adverse effect of perinatal nicotine exposure on heart development may suggest a new insight into prevention or treatment of fetal development of adult cardiac dysfunction. Although increasing evidence in animal models show a protective and beneficial effect of antioxidant treatment for cardiovascular dysfunction, there is insufficient evidence in humans to show a clear protective effect. Therefore, it is very important for our scientific research community to translate the animal studies into human clinical application.

## Supporting Information

S1 Fig Original blot image for Fig 5. Hearts were isolated from adult offspring that were prenatally exposed to saline control or nicotine along without or with NAC treatment. The original Western blot images of PKC_ε_ protein and GADPH protein were presented.
(PPTX)

S2 Fig Original blot image for Fig 6A. Hearts were isolated from adult offspring that were prenatally exposed to saline control or nicotine along without or with NAC treatment. The original Western blot images of p-GSK3β protein and GADPH protein were presented.
(PPTX)

S3 Fig Original blot image for Fig 6B. Hearts were isolated from adult offspring that were prenatally exposed to saline control or nicotine along without or with NAC treatment. The original Western blot images of total GSK3β protein and GADPH protein were presented.
(PPTX)
