# Supplementary material for: Protective Effect of Antenatal Antioxidant on Nicotine-Induced Heart Ischemia-Sensitive Phenotype in Rat Offspring
Source: PLoS One. 2016 Feb 26;11(2):e0150557. doi: 10.1371/journal.pone.0150557 (PMC4769226; doi:10.1371/journal.pone.0150557)
Supplement: S2 Fig — Hearts were isolated from adult offspring that were prenatally exposed to saline control or nicotine along without or with NAC treatment. The original Western blot images of p-GSK3β protein and GADPH protein were presented. (PPTX) [file pone.0150557.s002.pptx]

## Slide 1
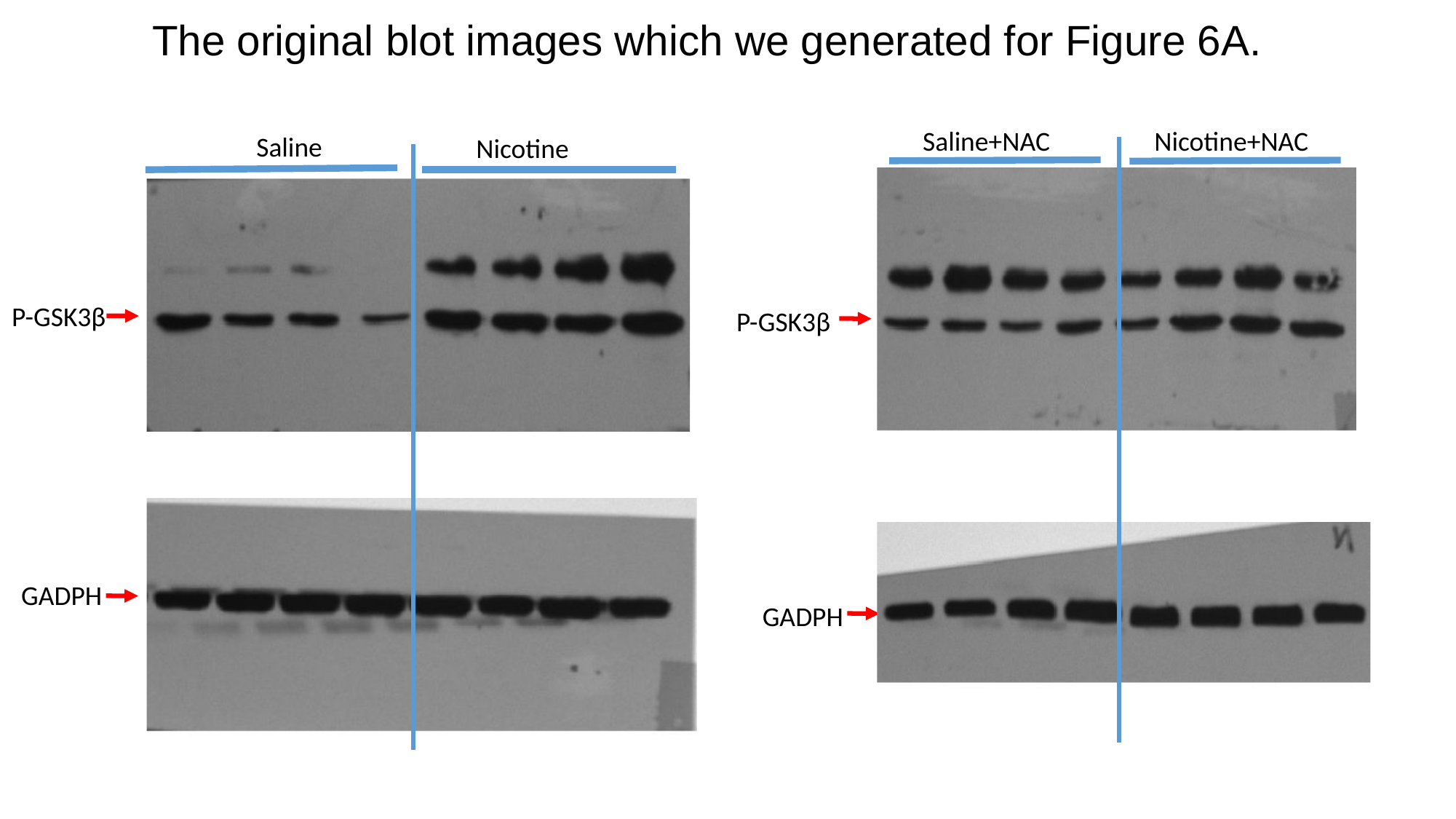

The original blot images which we generated for Figure 6A.
Saline+NAC
Nicotine+NAC
Saline
Nicotine
P-GSK3β
P-GSK3β
GADPH
GADPH
