# Supplementary material for: Protective Effect of Antenatal Antioxidant on Nicotine-Induced Heart Ischemia-Sensitive Phenotype in Rat Offspring
Source: PLoS One. 2016 Feb 26;11(2):e0150557. doi: 10.1371/journal.pone.0150557 (PMC4769226; doi:10.1371/journal.pone.0150557)
Supplement: S3 Fig — Hearts were isolated from adult offspring that were prenatally exposed to saline control or nicotine along without or with NAC treatment. The original Western blot images of total GSK3β protein and GADPH protein were presented. (PPTX) [file pone.0150557.s003.pptx]

## Slide 1
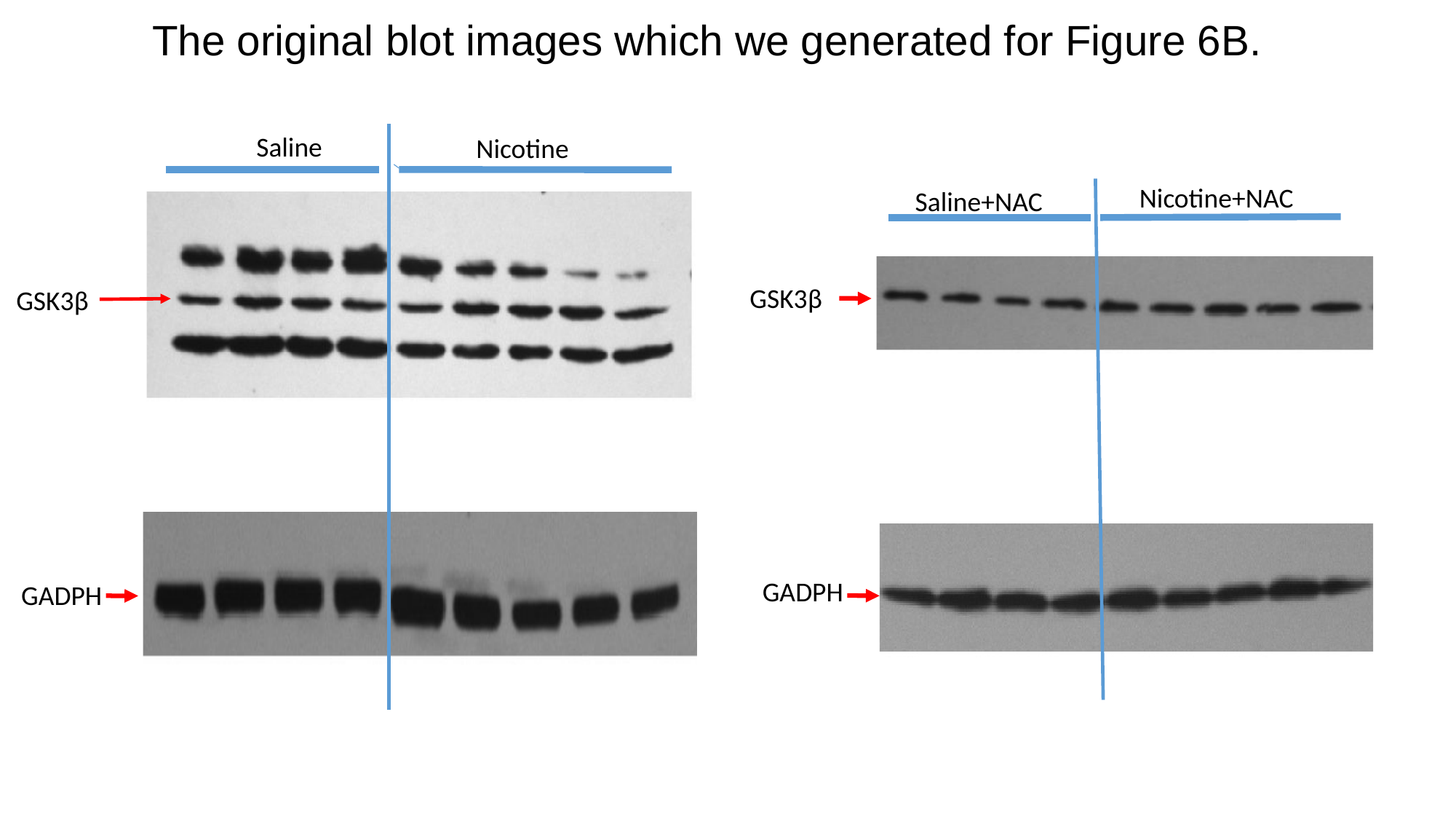

The original blot images which we generated for Figure 6B.
Saline
Nicotine
Nicotine+NAC
Saline+NAC
GSK3β
GSK3β
GADPH
GADPH
